# Stable gullies provide a suitable habitat for functional insects and reduce the threat of pests on crops in farmland of Northeast China

**DOI:** 10.1002/ece3.11686

**Published:** 2024-07-07

**Authors:** Haijun Zhang, Shaoliang Zhang, Chengbo Zhang, Ziliang Xiao, Pengke Yan, Muhammad Aurangzeib

**Affiliations:** ^1^ Northeast Agricultural University Harbin China

**Keywords:** functional insect, gully, interspecific association, Northeast China, plant diversity

## Abstract

Gullies with lower altitudes compared to the surrounding environment are widely distributed in farmland of the watershed and their numbers are still expanding. However, it is still unclear how these gullies regulate the functional insects in farmland. In this study, land use types combined with the herbaceous plant, herbicide application, soil moisture, topography and climatic factors during crop growth were considered to understand how gullies influence the dynamics of functional insects in farmland from a watershed (240 ha) of Northeast China. The primary findings demonstrate that the richness and abundance of functional insects are generally greatest in gullies, particularly in stable gullies, and decrease in the following order: forest belts, grasslands, and farmlands within the watershed. Notably, the ratios of beneficial insects to pests (BI/Pest) in terms of richness and abundance were lower in gullies before July but reversed after July, in comparison to farmland. Stable gullies exhibited higher BI/Pest abundance and diversity ratios than developing gullies. The richness and abundance of functional insects were higher in the middle sections of gullies compared to their heads and tails. Furthermore, the ratios of BI/Pest were generally lower in farmlands than in any gully position. Functional insect dynamics were mainly determined by season, followed by plant abundance and biomass in the gullies, and rarely by soil moisture in the both watershed and single gullies scales. Generally, the richness and abundance of functional insects in farmland were mainly influenced by gullies, especially influenced by the gully middle position. Insect composition in farmland influenced by stable gullies was stronger than by developing gullies, and stable gullies were more beneficial in reducing the threat of pests to crops in the farmland of the watershed.

## Graphical abstract

Our study indicated that stable gullies with higher plant diversity provide the best habitat for functional insects rather than forest belts and grassland in the watershed, especially during busy farming activities. The vigorous growth stage was one key period for pest control, and gully as a source of BI can significantly regulate the pests in farmland, further reducing the threat of pests to crops in this stage, especially in the position of middle stable gullies. In both the watershed and single gullies scales, functional insect dynamics were determined by season, followed by plant abundance and biomass in the gullies, and rarely by soil moisture. This provides guidance for the biological control of cropland pests in agricultural landscapes.

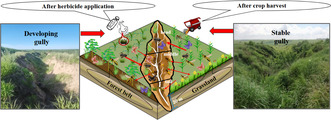

## 1 INTRODUCTION

Insect diversity as the main component of global biodiversity conservation programs has been given more attention in recent decades (Kremen & Merenlender, 2018; Phillips et al., 2019). In the agricultural systems, the abundance and biodiversity of insects including beneficial insects (BIs) and pests not only relate the global insect diversity, but also severely affect crop yield and quality (Brandt et al., 2017; Lyu et al., 2023). Notably, the control of pests and diseases in farmland by BI has been valued at over $400 billion annually worldwide (Costanza et al., 1997; Redhead et al., 2020). This also indirectly reduces the dosage application of pesticides, and further decreases environmental pollution (Cordoba‐Aguilar et al., 2021; Huang et al., 2020; Lyu et al., 2023). However, many reports indicated that the insects' abundance, especially the BI, was typically declining (Brooks et al., 2012; Ollerton et al., 2014; Powney et al., 2019), resulting in a decline of insect's service functions in the farmland (Oliver et al., 2015; Raven & Wagner, 2021). It is worth mentioning that semi‐natural habitats (SNH) are the key birthplaces of insects in farmland, and the critical refuges when chemical agents are used for controlling weeds and pests (Brandt et al., 2017; Garratt et al., 2017; Senapathi et al., 2017). SNH such as gullies, grasslands and forest belts, especially stable gullies, are widely distributed in farmland in the watershed, and have a lower altitude compared with farmland (Zhang et al., 2022; Zhang, Zhang, et al., 2023a). However, it is still unclear how these gullies influence the main functional insects' dynamics in farmland.

The distribution of insects is mainly influenced by vegetation diversity and biomass in SNH in agricultural landscapes (Filgueiras et al., 2019; Price, 1997). Higher plant diversity and biomass tend to provide more ecological niches for insects and influence the habitat conditions, thus further increasing insect diversity (Filgueiras et al., 2019; Geiger et al., 2009; Price, 1997). Also, insect diversity decreases with increasing distance between farmland and SNH (Feng et al., 2017; Knapp & Řezáč, 2015). This implies that insect distribution and dynamics are significantly influenced by the spatiotemporal patterns of vegetation, which may vary across different landscapes, necessitating further evidence. Studies also indicate that soil moisture was a key factor, and usually positively influences the insect abundance in different habitats (Zhang, Zhang, et al., 2023a). Additionally, insect diversity is commonly linked to temperature and precipitation, and temperature can also influence SNH gully formation by affecting snowmelt (Hawkins et al., 2003). Climate factors can also indirectly affect insect diversity by altering the quantity and quality of insect food resources (Njovu et al., 2021). Besides, anthropogenic disturbances, including herbicide application, mainly affect the population dynamics and growth of BI and pests, and force migration of some populations of BI (Schmidt et al., 2008). Farming activities such as plowing and harvesting can also cause cascading effects on functional insects due to depletion of resources locally (Geiger et al., 2009). All of these disturbances can force insects to migrate to adjacent, suboptimal habitats. Although the spatiotemporal pattern of insects is seriously affected by the vegetation, soil moisture, climate, and anthropogenic factors, the results may also differ in habitats. Especially for gullies with lower altitudes influencing the distribution of main functional insects may be different and lack report.

The factors that drive the spatiotemporal distribution of insects in different scales (small habitat and watershed scale) were not entirely consistent in agricultural landscapes (Zhang, Zhang, et al., 2023a). In the small habitat scale, complex and diverse habitats tend to maintain higher insect diversity due to higher plant diversity than simple habitats, and the competition between predator and prey also influence the size of insects population (Estrada‐Carmona et al., 2022; Zhang, 2014). Also, human disturbance can cause changes in habitat conditions, such as mowing, which reduces the diversity of insects (Hu et al., 2010; Zhang, Zhang, et al., 2023a). This implies that the changes in plant diversity, population competition and habitat conditions are determinants of the spatiotemporal dynamics of insects in small‐scale habitats. In the watershed scale, higher proportions of SNH maintained higher species diversity than lower proportions in agricultural landscapes, and varied from habitat to habitat (Estrada‐Carmona et al., 2022; González et al., 2017; Schmidt et al., 2005). This indicated that the spatiotemporal distribution of insects was determined by the distribution of vegetation resource in the watershed scale. In addition, the retention of grassland corridors could maintain the connectivity of fragmented landscapes, also utilized by different insects, and further reduce insect extinctions in the agricultural landscape (Wilson & Willis, 1975). Because the main driving forces of the spatiotemporal distribution of insects are not completely consistent in scales (Neokosmidis et al., 2018), it is important to consider the multiple scales to understand how landscape patterns influence the distribution of insects. Meanwhile, interspecific associations had scale dependence, with species‐pairs showing negative correlations at smaller scales and positive correlations at watershed scales (Hortal et al., 2010; Liu et al., 2018; Zhang, Zhang, et al., 2023a). Currently, these associations are predominantly applied to communities of plants (Jin et al., 2022; Liu et al., 2018; Lu et al., 2020), while in insect ecology it can also clearly reveal the interrelationships between species within a community (Zhang et al., 2003; Zhang, Zhang, et al., 2023a). However, interspecific associations of functional insects in gully habitats have not been well reported.

It is noteworthy that gullies (including stable and developing gullies) are widely distributed in farmland in the Mollisols region of Northeast China. Stable gullies usually represent that gully head, bank and bottom are stable (no movement) and has relatively better vegetation recovery, while developing gullies show that gully size is increasing and has poorer vegetation recovery during different stages of succession (Zhang, Guo, et al., 2023b; Zhang, Zhang, et al., 2023a). These gullies occupy a large proportion of agricultural land and their number progressively increases each year (Zhang et al., 2022; Zhang, Guo, et al., 2023b). However, it is still not clear (1) how these gullies affect the distribution of functional insects in farmland, especially gully types; (2) how the differences in gully positions affect the distribution of the main functional insects; and (3) what are the key factors affecting the main functional insects and the response at different scales. Thus, our study investigated the spatiotemporal dynamics of main functional insects influenced by herbicide application, plant diversity, soil moisture, topography, and climatic factors during crop growth in the watershed and the single gully scales, respectively. This study aims to deeply understand the influence of gullies in farmland on the spatiotemporal dynamics of main functional insects in agricultural watersheds, to identify key influencing factors, and to guide the regulation of pests and diseases in farmland by multiple scales.

## 2 MATERIALS AND METHODS

### 2.1 Study area

**FIGURE 1:**
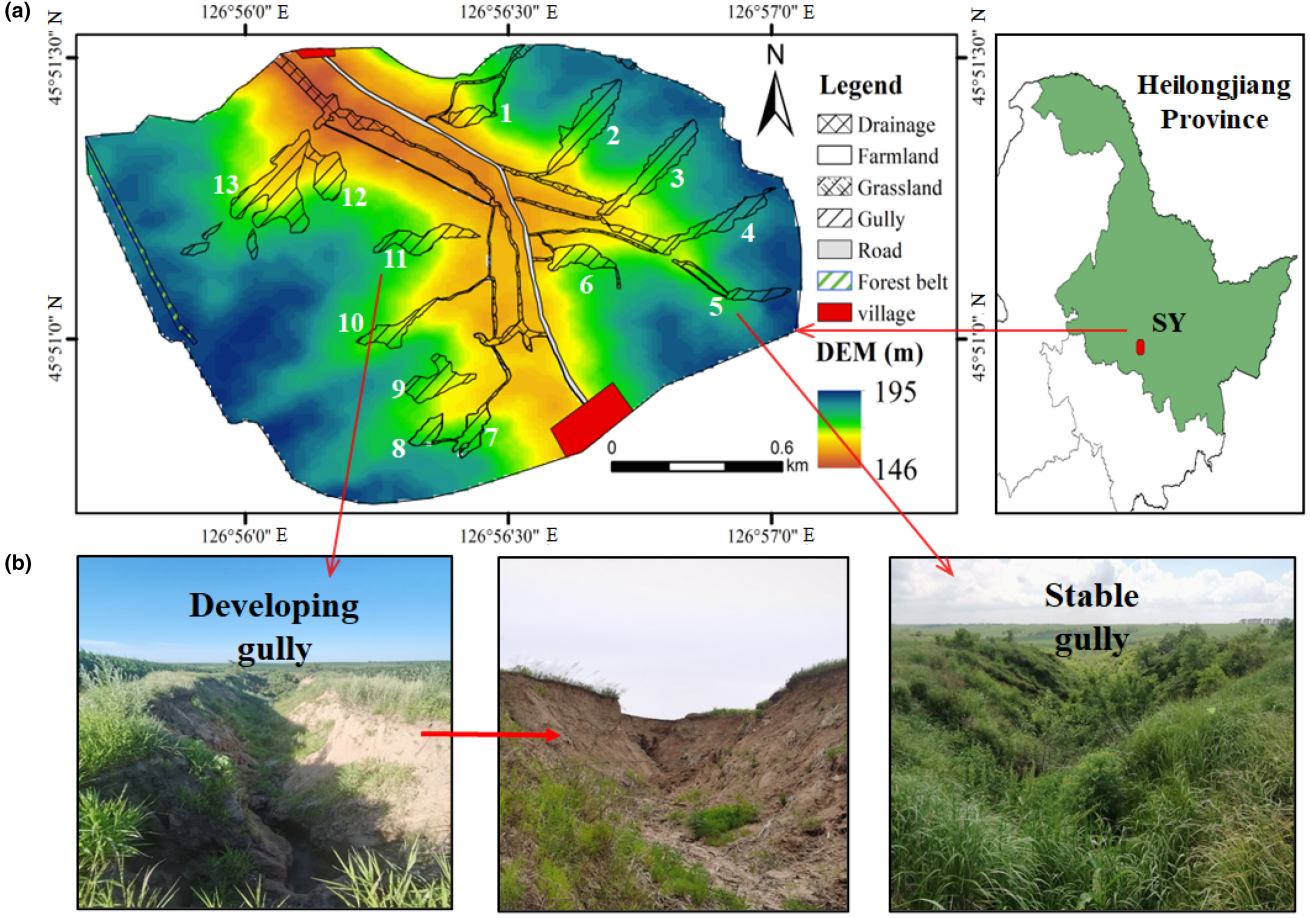
Locations of gullies investigated in the Mollisols of Northeast China. SY represents the location of Shang Yan village in Northeast China (Sub‐figure‐a). The gullies were named by continuous numbers. Sub‐figure‐b represents the type of gully in the study area. The detailed information of gullies was described in Table 1.

The study watershed (240 ha) is situated in Shang Yan Village, Harbin City, Heilongjiang Province, Northeast China (Figure 1a). This region is located in the continental monsoon climate zone, and hot and rain happen in the same period. Over the past two decades, the average temperature has been 3.6°C, with annual precipitation at 540 mm. The mean temperature peaks in July–August (average 25.5°C) and reaches its lowest in January (average −18°C). Most SNH is covered with natural vegetation beginning in early April, and mostly species bloom in July–August (Table S1) while withering in September–October. Soil development commenced during the Quaternary age, organic matter is relatively rich, and its soil type is categorized as Mollisols based on the US Soil Taxonomy (USST) (Zhang et al., 2014, 2021).

In the watershed, farmland constitutes more than 80 percent of the area, and there were little difference between the management of different farmlands. Maize (*Z. mays*) has been the dominant over crop in the past few decades, including 2021 and 2022. The SNH includes gullies, forest belts, and grasslands, occupy 20% of the watershed area, and their dominant vegetation types are described in detail in Table S1. Thirteen gullies, primarily located in the midst of farmland (Figure 1), are detailed in Table 1. Insects were measured in farmlands, gullies, forest belts, and grasslands, focusing on four specific gullies for an in‐depth analysis. The second and third gullies were identified as the stable, whereas the eleventh and thirteenth were categorized as the developing (Table 1). Farmland began to be plowed in May, and weeds were controlled by herbicide (atrazine, 600–720 mL/acre) in June, followed by fertilization from late June to middle July. Maize blooms in July, and harvests in middle October. No significant pest or disease outbreaks were reported on maize fields during the two‐year study period, and insecticides were not applied by farmers.

**TABLE 1:** Description of gullies and soil properties investigated in the studied gullies.

Gully number	Gully types^a^	Gully head area (m^2^)	Gully middle area (m^2^)	Gully tail area (m^2^)	Distance to farmland (m)	Length (m)	Total area (m^2^)	Depth (m)
1	Stable	1399	2026	1453	2.1	117	4878	14
2*	Stable	6213	7948	6011	3.8	360	20,172	20
3*	Stable	5453	7071	4999	2.1	369	17,523	16
4	Development	4911	5509	4909	3.4	445	15,329	15
5	Stable	1386	1693	1397	3.1	154	4476	11
6	Development	3042	3086	2554	2.4	159	8683	12
7	Development	2246	2596	2046	3.6	194	6889	13
8	Stable	1363	1956	1163	3.4	126	4482	12
9	Stable	3677	4782	2856	2.1	184	11,315	16
10	Stable	2315	3465	1916	3.4	246	7696	17
11*	Development	2974	4759	3194	2.8	318	10,906	15
12	Stable	3356	3964	3073	3.2	162	10,393	17
13*	Development	8669	12,021	8269	3.3	347	28,960	17

^a^ Gully types: Stable gullies represent gully head is stable (no movement) and has a relatively completed vegetation recovery. Developing gullies represent gully head is moving with a poor vegetation recovery (Zhang, Zhang, et al., 2023a). The gullies marked by “*” were investigated in detail.

### 2.2 Sampling and measurement methods

#### 2.2.1 Hanging traps

The insects were surveyed using the higher capture efficiency hanging traps method (Figure S1). Three sample points were evenly arranged in the main habitats of gullies and surrounding farmland, forest belt and grassland, each sample point hanging a set of fluorescent bowls (including the yellow, white and blue). Three replications were set up for each sample point, and a total of 27 fluorescent bowls for each habitat. The trap bowls, measuring 11.4 cm diameter and 6 cm high, were initially coated with fluorescent paint in yellow, white and blue. Subsequently, the bowls were mounted on bamboo poles, which were of a height comparable to the surrounding vegetation at the sampling site, and these poles were inserted approximately 10 cm into the ground. Each bowl was filled to one‐third of its volume with saturated saltwater, to which several drops of detergent (Liby) were added to reduce the surface tension of solution. In this method, fluorescent bowls simulate plant flowering, and different colored bowls were used as a complement to capture insects more effectively (Wang et al., 2017). These fluorescent bowls were randomly distributed at each sample point with a distance of at least 5 m between each sample point. Insect samples were collected after 48 h in the field (Westphal et al., 2008), and once a month.

Investigations were conducted during key agricultural phases: early vegetative growth coinciding with herbicide application (5 June−11 June), late vegetative growth (22 June−28 June), flowering (14 July−20 July), maturity (1 September−5 September), and harvest (10 October−15 October).

#### 2.2.2 Pitfall traps

Traps were utilized to capture functional insects with limited mobility, predominantly Carabid beetles. The placement of these traps mirrored the methodology described for Hanging traps, with a bowl positioned in each sampling point and three replicates arranged at each sample point. Each habitat contained 9 bowls, with the rim of each trap bowl set slightly below soil surface. The traps were assessed for functional insects after 1 week of trapping and repeated monthly.

#### 2.2.3 Insect classification and identification

The collected insects were mainly classified as pests, pollinators and natural enemies, and pollinators and natural enemies were collectively referred to as BIs in this study. Insects that directly eat the vegetative and reproductive organs of the crop and cause actual or potential harm to the growth and yield of the crop are defined as pests, conversely as non‐pest herbivores. The judgment of insect impacting the local crops was determined though consultation with relevant experts, references and book descriptions (Ge, 2008). Insects that can prey on pests that eat the crop (natural enemies) and the pollinators that pollinate crops and wild plants are defined as BI (Ge, 2008; Wang et al., 2017). Functional insects were identified mainly by referring to relevant books (Zhang & Li, 2011), and morphological features such as differences in antennae, mouthparts and wings were observed using a NOVEL NSZ‐608 T binocular stereomicroscope. The richness and abundance of all species were recorded. The proportion of each functional insect identified so far in this study (Figure S5).

#### 2.2.4 Measurements of plant and soil moisture

Abundance and richness of herbaceous plants were calculated using a plot (1 m × 1 m), which was arranged around the trap bowls. Plant samples were collected to the laboratory for killing (105°C), and biomass was measured by oven drying to constant weight (75°C), with the same sampling period as insect sampling.

The time‐domain reflectance (TDR350 USA) (dual probes, 20 cm) method was used to measure soil moisture (0–20 cm). Five sample points were evenly distributed in the gullies (head, middle and tail), farmlands, forest belts and grassland corridors, and three replicates were set up for each sampling point. Soil moisture was measured in the same period as that for insect sampling.

Climate data (precipitation, temperature) are from the China Meteorological Data Network (http://data.cma.cn).

### 2.3 Data analysis

The Appendix Supplementary Data provides a detailed description of the methods used to calculate alpha‐diversity and interspecies association indices for functional insects.

### 2.4 Statistical analyses

The normality of the main functional insect's data distribution was assessed using the Shapiro–Wilk test, and variance homogeneity was evaluated via Levene's test. The Spearman's rank correlation coefficient to determine the correlation strength among main functional insects. Differences among functional insects across various locations and stages within watersheds and single gullies were analyzed using one‐way ANOVA and the least significant difference (LSD) test. The alpha‐diversity indices of main functional insects were computed in R 4.3.2, and the β‐diversity of different habitats was expressed by NMDS analysis. Spatiotemporal patterns of functional insects were mapped using the inverse distance weighted method in ArcGIS 10.8. Generalized linear models (GLMs) were used to evaluate the effects of different predictor variables on species richness and abundance in R 4.3.2. Structural equation modeling (SEM) for assessing the influencing factors on insects was performed using AMOS 22. All figures were created with Origin 2020, and data were expressed as the mean ± SD.

## 3 RESULTS

### 3.1 Dynamics of functional insects richness and abundance in the watershed

**FIGURE 2:**
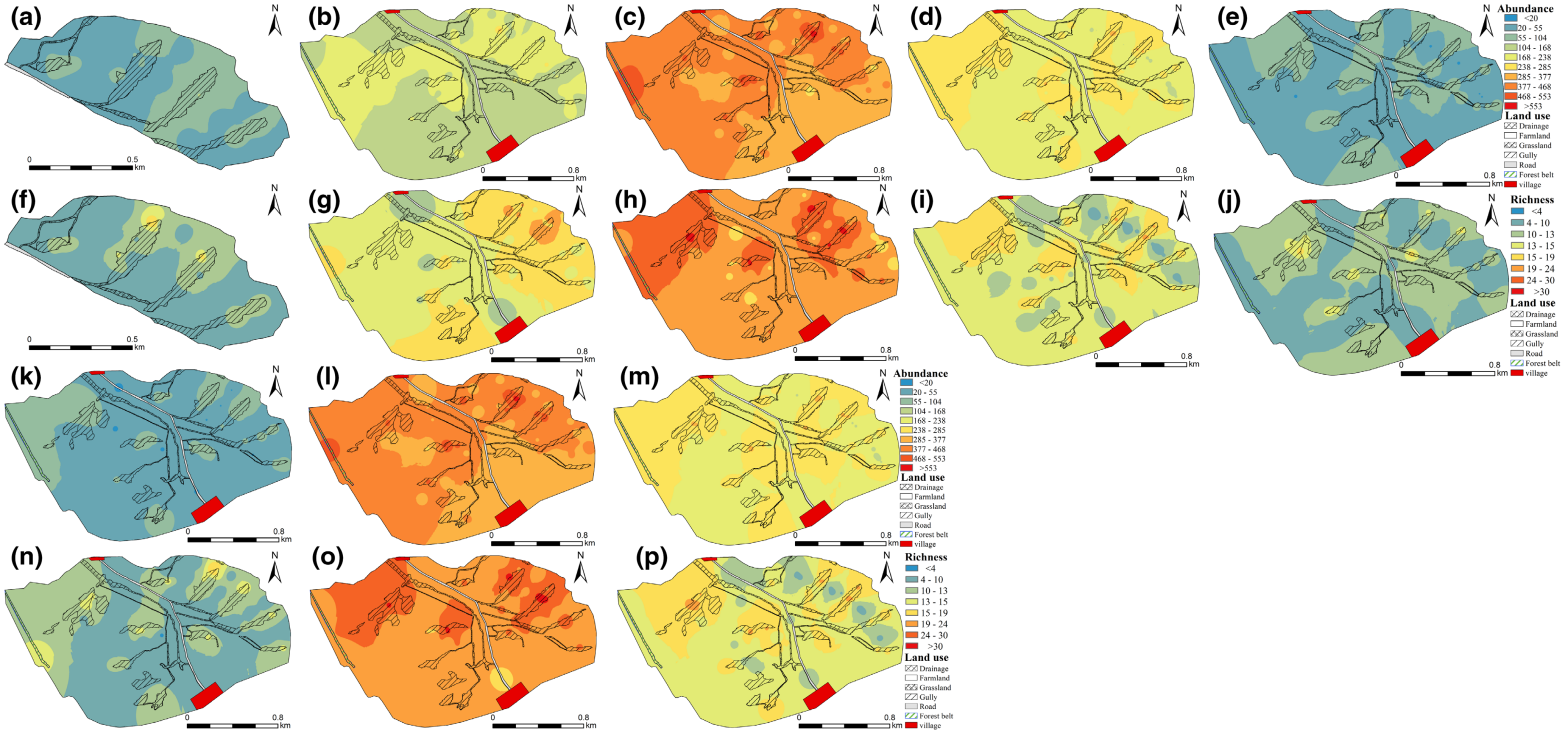
Spatiotemporal distribution of insects in the watershed. Sub‐figures of a, b, c, d and e represent of spatial distribution of the abundance of insects on 11 June, 28 June, 20 July, 5 September and 15 October in 2021, respectively. Sub‐figures of f, g, h, i and j represent of spatial distribution of the richness of insects on 11 June, 28 June, 20 July, 5 September and 15 October in 2021, respectively. Sub‐figures of k, l and m represents of spatial distribution of the abundance of insects on 11 June, 20 July and 24 August in 2022, respectively. Sub‐figures of n, o and p represents of spatial distribution of the richness of insects on 11 June, 20 July and 24 August in 2022, respectively.

**FIGURE 3:**
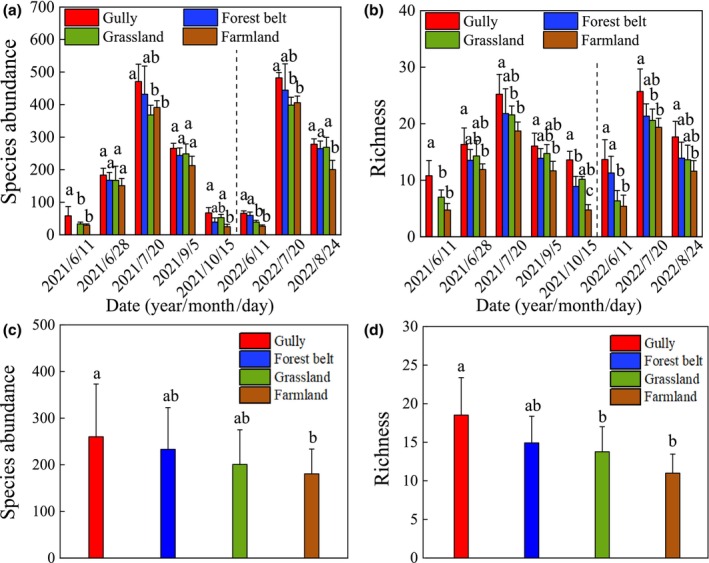
Differences of the abundance and richness of insects between habitats in the watershed. Sub‐figures of a and b represent the differences of insects between habitats during the same period, and sub‐figures of c and d represent the difference between habitats during the entire sampling stages. The different lowercase letters indicate a significant difference within the same variable (*p* < .05).

The richness and abundance of insects gradually increased to peak value from spring to the bloom stage of crop firstly, and then gradually decreased with time in the watershed (Figures 2 and 3a,b). Compared to farmland, insect abundance and richness were 1.23 and 1.37 times greater in gullies in vegetative and maturity growth stages of the crop and were 1.2 and 1.34 times greater during crop blooming in gullies (*p* < .05) (Figure 3a,b). In addition, the forest belt had a lower insect abundance and richness than in gullies, but was still 1.1 and 1.13 times higher than in farmland (Figure 3a,b). And, the mean insect abundance of in near villages/roadsides was about 1.32–1.62 times lower than in farmland (Figure 2a–e).

Notably, insect dynamics displayed consistent patterns following herbicide application and crop harvesting, with a tendency for insects to aggregate along gullies and grassland corridors (Figure 2a,e,f,j,k,n). The insect richness and abundance were 2.6 and 2.4 times higher in gullies compared to farmland (Figure 3a,b). Across all crop growth stages, both the insect richness and abundance peaked in gullies, succeeded by forest belts, grasslands, and farmlands, with richness notably higher in gullies than in grasslands and farmlands (Figure 3c,d). Also, β‐diversity of species varied among habitats in the watershed, especially in gullies and farmland (Figure S4).

**FIGURE 4:**
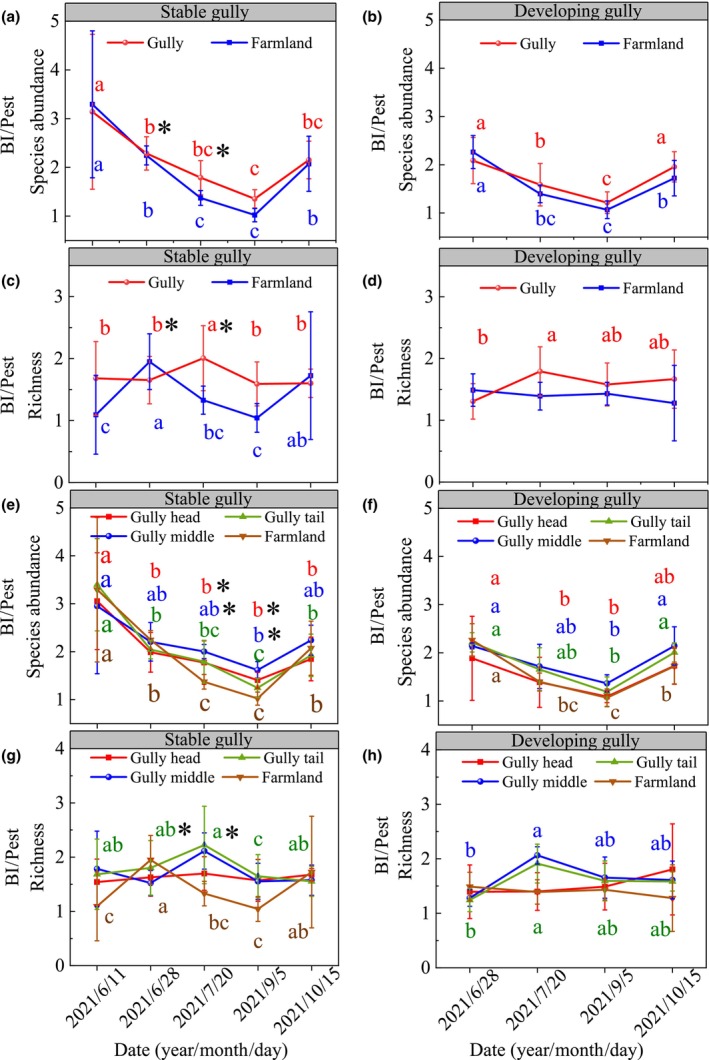
Dynamics of the ratios of the abundance and richness of BIs to pests (BI/Pest) in the watershed and single gullies scale. Sub‐figures of a, b, c, and d represent the BI/Pest dynamics of abundance in stable gully, abundance in developing gully, richness in stable gully, richness in developing gully in the watershed scale, respectively. Sub‐figures of e, f, g and h represent the BI/Pest dynamics of abundance in stable gully, abundance in developing gully, richness in stable gully, richness in developing gully in the single gullies scale, respectively. *Represents higher level of stable gullies than developing gullies in the same position of the same period (*p* < .05).

The dynamics of abundance ratios of BI/Pest in gullies and farmland decreased from spring to September and then increased. Both the richness and abundance ratios of BI/Pest were lower in gullies before July but were opposite after July compared with farmland, and the stable gullies typically had a higher richness and abundance ratios of BI/Pest than the developing gully (Figure 4a–d).

### 3.2 Dynamics of functional insects richness and abundance in single gullies

**FIGURE 5:**
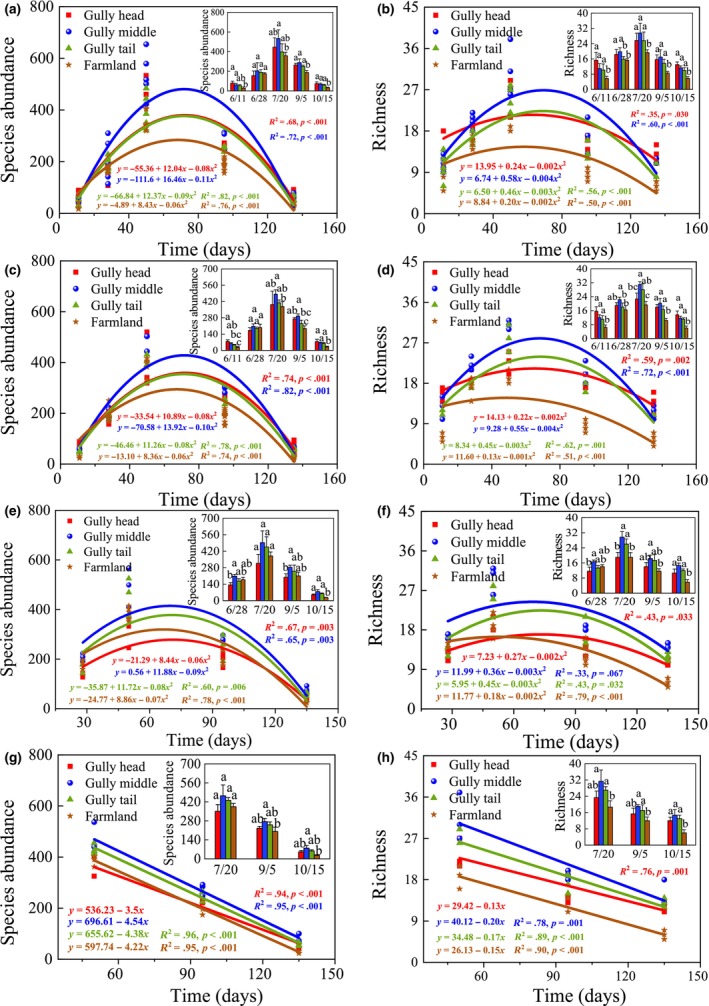
Dynamics of abundance and richness of insect with time in the single gullies. Sub‐figures of a, b and c, d represent the 2nd and 3rd stable gullies, and sub‐figures of e, f and g, h represent the 11th and 13th developing gullies, respectively, as described in detail in Table 1. The different lowercase letters indicate a significant difference in the abundance and richness of insects between locations during the same period (*p* < .05).

The richness and abundance of insects increasing with time can be modeled as quadratic functions in all positions of gullies and their adjacent farmlands in single gullies (*p* < .05) (Figure 5a–f). Their dynamics peaked in early August before declining. It is noteworthy that the richness and abundance ratios of BI/Pest were mostly lowest in farmland than in all gully positions, while the abundance ratios of BI/Pest in gully middle was highest and the richness ratios of BI/Pest in gully head was lowest than other gully position for both stable and developing gullies (Figure 4e–h).

Following herbicide application, insect abundance and richness reached their peak in gully head in stable gullies, being 2.7 and 2.4 times greater than in farmland, respectively (*p <* .05) (Figure 5a–d), while in developing gullies, the highest values were observed in gully middle (Figure 2). Notably, the disparity in insect richness and abundance among gullies and farmlands was most pronounced immediately after herbicide application compared to other phases of crop growth. Also, in both stable and developing gullies, insects' richness and abundance were predominantly greatest in gully middle, followed by gully tail, and gully head (Figure 5).

### 3.3 Functional insects interspecific association in the watershed

**FIGURE 6:**
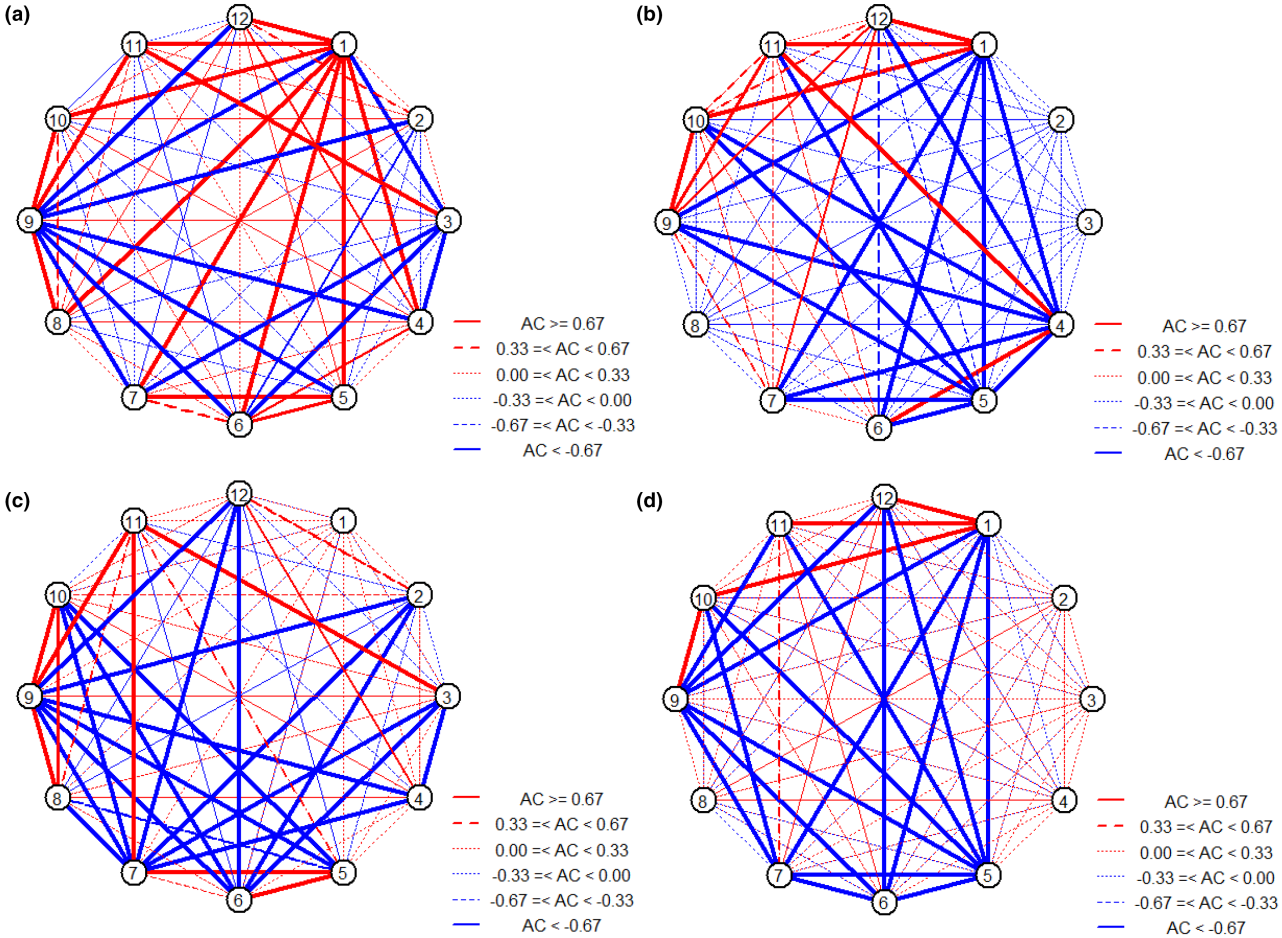
ACs between main insect species. Sub‐figure a and b represents the interaction of insect species after herbicide application and during the bloom stage in the watershed scale, respectively. Sub‐figure c and d represents the interaction of insect species after herbicide application and during the bloom stage in the single gullies, respectively. Sub‐figure a and c the serial number from 1 to 12 represents the insect species of Formicidae, Delphacidae, Chrysomelidae, Pyralidae, Bibionidae, Cicadidae, Apidae, Muscidae, Acridoidea, Carabidae, Syrphidae, Ceratopogonidae. Sub‐figures b and d the serial number from 1 to 12 represents of Chrysomelidae, Gryllidae, Formicidae, Muscidae, Delphacidae, Acridoidea, Pyralidae, Meloidae, Apidae, Scarabaeidae, Panorpidae, Carabidae.

The criteria for selecting the top 12 functional insects resulted in importance values that exceeded 2% after herbicide application. A variance ratio (VR) of 1.81 (>1), whereas the statistic value (*W* = 75.96) did not fall within the *χ* ^2^ critical value (Table 2). This indicates a significantly positive in the overall community of functional insects. Chi‐square test indicated a significantly positive between 6 species‐pairs (9.1%), while a negative between 18 species‐pairs (27.3%) (Figure S2a). Similarly, the association coefficients (ACs) indices revealed a significantly positive between 14 species‐pairs (21.2%), while a negative between 11 species‐pairs (16.7%) (Figure 6a). Furthermore, the Spearman's rank correlation coefficient (SRCC) highlighted a significantly positive between 10 species‐pairs, and a negative between 2 species‐pairs (Table 3). This indicates a predominantly significantly positive association between species‐pairs, consistent with the AC results.

**TABLE 2:** The overall interspecific associations of main species groups in insect communities at different scales under different stages.

Scale	Time	Variance ratio (VR)	Statistic (W)	Df	χ^2^ critical value (χ^2^_0.95_, χ^2^_0.05_)	Verification results
watershed	6/11	1.81	75.96	41	(26.51, 55.76)	Significantly positive association
	7/20	2.35	218.6	92	(69.13, 113.14)	Significantly positive association
Single gully	6/11	1.07	23.51	21	(11.59, 32.67)	Not significantly positive association
	7/20	1.32	52.98	39	(26.51, 55.76)	Not significantly positive association

**TABLE 3:** Correlation coefficient of spearman rank and the niche overlap between main species after herbicide application in the watershed.

	1	2	3	4	5	6	7	8	9	10	11	12
1		.09	−.15	.09	−.44**	−.10	.07	−.11	.20	.18	−.14	.23
2	.62		−.06	−.10	−.25	−.45**	−.30	.14	.15	.17	.16	.28
3	.65	.57		.17	.40**	.44**	.14	.29	.34*	.08	.09	−.04
4	.53	.46	.61		.20	.44**	.12	.31*	.01	.31*	−.03	.51**
5	.24	.22	.58	.36		.54**	.66**	.03	−.03	−.23	.13	.04
6	.52	.23	.63	.66	.61		.45**	.15	−.11	.01	−.15	−.02
7	.65	.33	.61	.50	.53	.65		−.16	−.03	−.25	−.03	.24
8	.50	.54	.63	.58	.34	.43	.32		.16	.63**	.25	.17
9	.81	.74	.77	.54	.39	.44	.58	.64		.22	.05	.06
10	.56	.52	.56	.60	.25	.36	.29	.78	.62		−.21	.29
11	.35	.46	.50	.41	.38	.22	.35	.52	.45	.30		.05
12	.31	.51	.47	.68	.15	.21	.34	.41	.40	.50	.55	

*Note*: The top right‐hand corner represents the spearman rank correlation and the lower left‐hand corner represents the niche overlap. * and ** represent the correlation was significant at the *p* < .05 and *p* < .01 levels (two‐tailed). Bold text represents significant correlation. The serial number from 1 to 12 represents of Formicidae, Delphacidae, Chrysomelidae, Pyralidae, Bibionidae, Cicadidae, Apidae, Muscidae, Acridoidea, Carabidae, Syrphidae, Ceratopogonidae.

The overall communities of functional insects showed a similar pattern of significantly positive in the flowering and after herbicide application in the watershed (Table 2). Chi‐Square test indicated a significantly positive between 6 species‐pairs (9.1%), and a negative between 4 species‐pairs (6.1%) (Figure S2b). The AC indices revealed a significantly positive between 9 species‐pairs (13.6%) and a negative between 15 species‐pairs (22.7%) (Figure 6b). Additionally, SRCC highlighted a significantly positive between 21 species‐pairs and a negative between 8 species‐pairs (Table 4).

**TABLE 4:** Correlation coefficient of spearman rank and niche overlap between main species during the bloom stage in the watershed.

	1	2	3	4	5	6	7	8	9	10	11	12
1		−.19	.05	.00	.18	.14	−.36**	−.05	−.22*	−.10	−.23*	−.04
2	.86		.20	.08	−.24*	.40**	.10	.18	.22*	.17	.24*	−.07
3	.86	.93		.54**	.12	.21*	.15	−.04	.36**	.39**	.19	.07
4	.86	.92	.95		−.07	.31*	.18	−.05	.53**	.44**	.29**	.07
5	.88	.87	.89	.87		.16	−.39**	−.13	−.17	−.14	−.26*	−.35**
6	.85	.93	.89	.90	.88		−.24*	.02	.28**	.31**	−.05	−.15
7	.61	.74	.74	.76	.62	.64		.10	.31**	.23*	.60**	.35**
8	.85	.93	.88	.88	.86	.88	.71		−.12	−.12	.07	.06
9	.70	.84	.85	.88	.73	.82	.78	.77		.69**	.44**	.09
10	.71	.83	.84	.85	.72	.82	.72	.76	.92		.25*	.26*
11	.60	.74	.72	.73	.60	.64	.79	.70	.75	.69		.17
12	.57	.59	.59	.59	.48	.51	.63	.61	.54	.60	.54	

*Note*: The top right‐hand corner represents the spearman rank correlation and the lower left‐hand corner represents the niche overlap. * and ** represent correlation was significant at the *p* < .05 and *p* < .01 levels (two‐tailed). Bold text represents significant correlation. The serial number from 1 to 12 represents of Chrysomelidae, Gryllidae, Formicidae, Muscidae, Delphacidae, Acridoidea, Pyralidae, Meloidae, Apidae, Scarabaeidae, Panorpidae, Carabidae.

### 3.4 Functional insects interspecific association in single gullies

After herbicide application, the VR was recorded at 1.07, and the W statistic was found to be within the χ^2^ critical value (Table 2). This result suggests that the overall community composition of functional insects did not demonstrate a significantly positive. Chi‐Square test revealed a significantly positive between 10 species‐pairs (15.2%) and a negative between 6 species‐pairs (9.1%) (Figure S2c). In addition, the AC index indicated a significantly positive between 8 species‐pairs (12.1%) and negative between 19 species‐pairs (28.8%) (Figure 6c). SRCC indicated a significantly positive between 4 species‐pairs and a negative between 5 species‐pairs (Table 5). Notably, the species–pairs association was not significantly positive in the flowering and after herbicide application (Table 2). Chi‐square test highlighted a significantly positive between 4 species‐pairs (6.1%) and a negative between 3 species‐pairs (4.5%) (Figure S2d). Moreover, the AC index showed a significantly positive between 4 species‐pairs (6.1%) and a negative between 17 species‐pairs (25.8%) (Figure 6d). SRCC indicated a significantly positive between 9 species‐pairs, and negative between 1 species‐pairs (Table 6).

**TABLE 5:** Correlation coefficient of spearman rank and niche overlap between main species after herbicide application in single gullies.

	1	2	3	4	5	6	7	8	9	10	11	12
1		−.04	−.20	.03	−.70**	−.07	.11	−.20	.10	.12	−.35	.16
2	.53		.20	.25	−.22	−.36	−.38	.21	.20	.44*	.05	.55**
3	.69	.67		−.05	.26	−.02	−.16	.41	.33	.10	.27	−.03
4	.65	.62	.65		.00	.04	−.20	.27	.01	.35	.05	.49*
5	.30	.19	.53	.39		.19	.32	−.17	−.23	−.47*	.28	−.19
6	.65	.27	.55	.65	.57		.09	−.01	−.70**	−.19	−.23	−.31
7	.79	.30	.64	.55	.52	.68		−.67**	−.27	−.66**	.09	.12
8	.59	.59	.79	.65	.36	.50	.36		.21	.67**	.28	.00
9	.79	.73	.79	.64	.37	.43	.62	.72		.25	.00	.17
10	.63	.65	.67	.69	.27	.40	.34	.83	.69		−.08	.21
11	.33	.36	.61	.49	.40	.25	.43	.55	.40	.43		.25
12	.38	.66	.62	.71	.20	.24	.37	.43	.49	.55	.69	

*Note*: The top right‐hand corner represents the spearman rank correlation and the lower left‐hand corner represents the niche overlap. * and ** represent the correlation was significant at the *p* < .05 and *p* < .01 levels (two‐tailed). Bold text represents significant correlation. The serial number from 1 to 12 represents represent the same insect species as in Table 3.

**TABLE 6:** Correlation coefficient of spearman rank and niche overlap between main species during the bloom stage in single gullies.

	1	2	3	4	5	6	7	8	9	10	11	12
1		.02	.17	.19	.03	.23	−.13	−.02	.18	.17	.11	−.10
2	.88		.20	−.41**	.00	.56**	−.17	.24	−.25	−.03	−.05	−.16
3	.89	.94		.47**	.31*	.33*	−.09	−.17	.22	.38*	−.03	−.19
4	.89	.91	.96		.20	.08	.12	−.26	.35*	.32*	.08	−.15
5	.85	.92	.93	.92		.40*	−.03	.00	.30	.25	.00	−.26
6	.84	.93	.89	.87	.91		−.28	−.04	.16	.30	−.20	−.19
7	.74	.76	.77	.81	.77	.67		.08	.21	.12	.34*	.12
8	.85	.95	.89	.88	.89	.85	.75		−.23	−.17	.04	.11
9	.84	.83	.87	.89	.87	.81	.84	.81		.60**	.23	.03
10	.83	.85	.88	.87	.86	.85	.75	.81	.92		.21	.03
11	.78	.81	.79	.81	.75	.70	.79	.77	.80	.78		.19
12	.73	.77	.76	.75	.69	.65	.69	.79	.71	.70	.74	

*Note*: The top right‐hand corner represents the spearman rank correlation and the lower left‐hand corner represents the niche overlap. * and ** represent correlation the significant at the *p* < .05 and *p* < .01 levels (two‐tailed). Bold text represents significant correlation. The serial number from 1 to 12 represents represent the same insect species as in Table 4.

## 4 DISCUSSION

### 4.1 Dynamics of functional insects in the watershed

Insect diversity was not only determined by the abundance, diversity and biomass of plants, but was also determined by the environmental conditions of micro‐landscapes in SNH (Ramzan et al., 2020; Zhang, Zhang, et al., 2023a). Gullies, characterized by a lower altitude relative to surrounding farmland, are widely distributed in farmland and typically have a complicated plant community under special micro‐landscape and microclimate (Zhang, Guo, et al., 2023b; Zhang, Zhang, et al., 2023a). Thus, these gullies affecting insects could be different from other SNH in farmland. In this study, both the richness and abundance of functional insects peaked in gullies, particularly in stable gullies, succeeded by forest belts, grasslands, and farmlands. Previous publications indicated that plant diversity is greater in gullies compared to the forest belts and farmlands, while the biomass was not, as well as the plant diversity typically exhibits a positive correlation with insect diversity (Price, 1997; Zhang, Zhang, et al., 2023a). Thus, stable gullies with higher plant diversity provide a better habitat for insects rather than forest belts and farmland in the watershed.

It is noteworthy that the ratios of BI/Pest can be used to reflect the balance of BI and pests in the farmland and understand what is the degree of pests that threatening crops (Zhang, Zhang, et al., 2023a). In this study, the richness and abundance ratios of BI/Pest were higher in gullies than in farmland after July and was typically greater in stable gullies than developing gullies (Figure 4a–d). Also, compared with other crop growth stages, the effect of gullies on the richness ratios of BI/Pest was greater in the bloom stage (July) (Figure 4c,d). This can be attributed to the fact that plant diversity and biomass were higher in the peak blooming stage of the crop than in other stages in gullies (Table S1). Thus, the plant's growth in gullies, especially during the growth stages of blooming when plants have relatively higher biomass, such as *Saussurea japonica* and *Aster hispidus*, which can feed more BI (Table S1, Figure S3), further improve the crop pollination and reduce the threat of pest on crops in this agricultural watershed.

#### 4.1.1 Insect diversity influenced by herbicides

**FIGURE 7:**
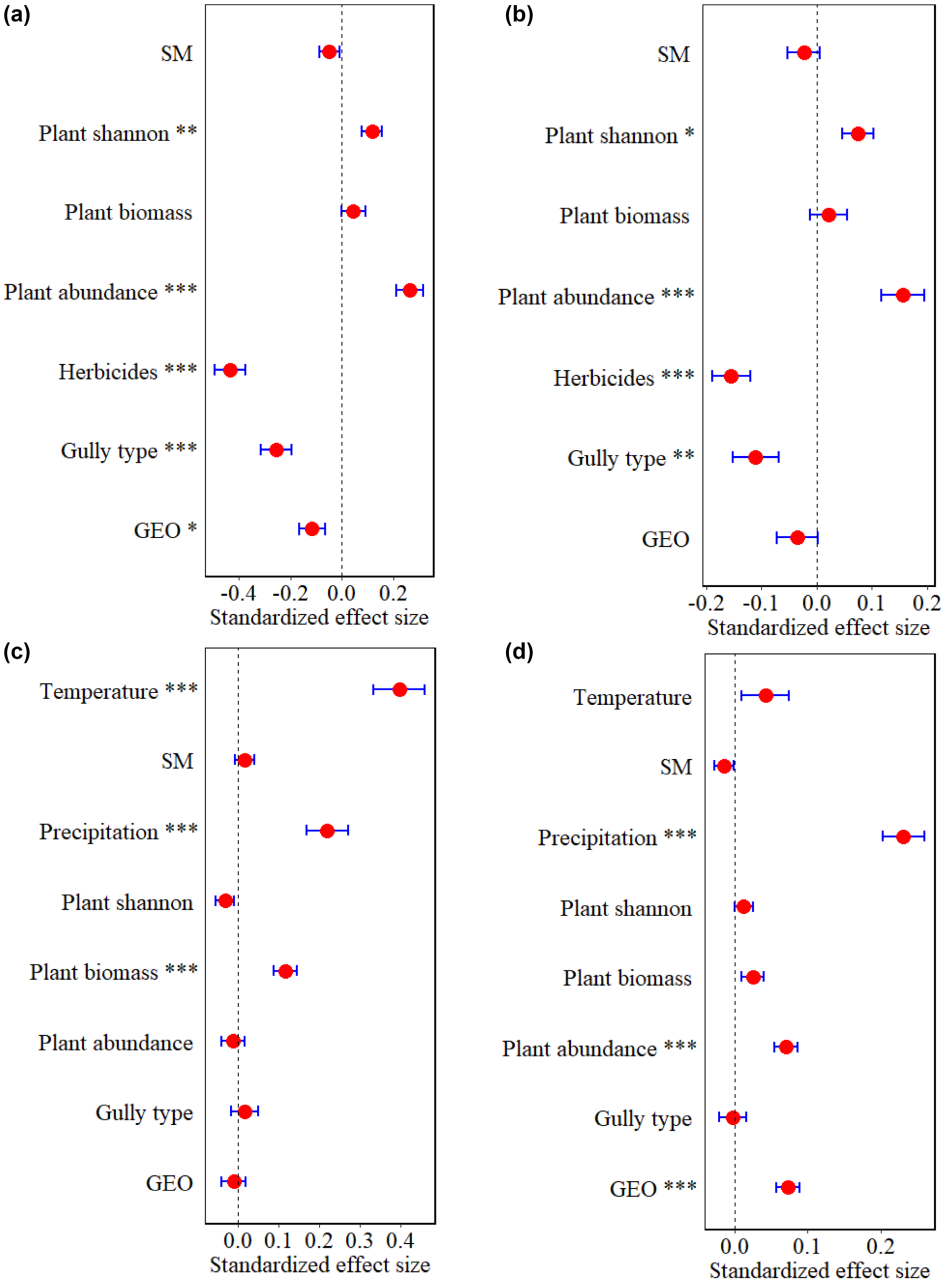
GLMs to test the effects of environmental factors on insect abundance (a, c) and richness (b, d). Sub‐figure a, b represents the after herbicides application, respectively, and c, d represents the entire period.

**FIGURE 8:**
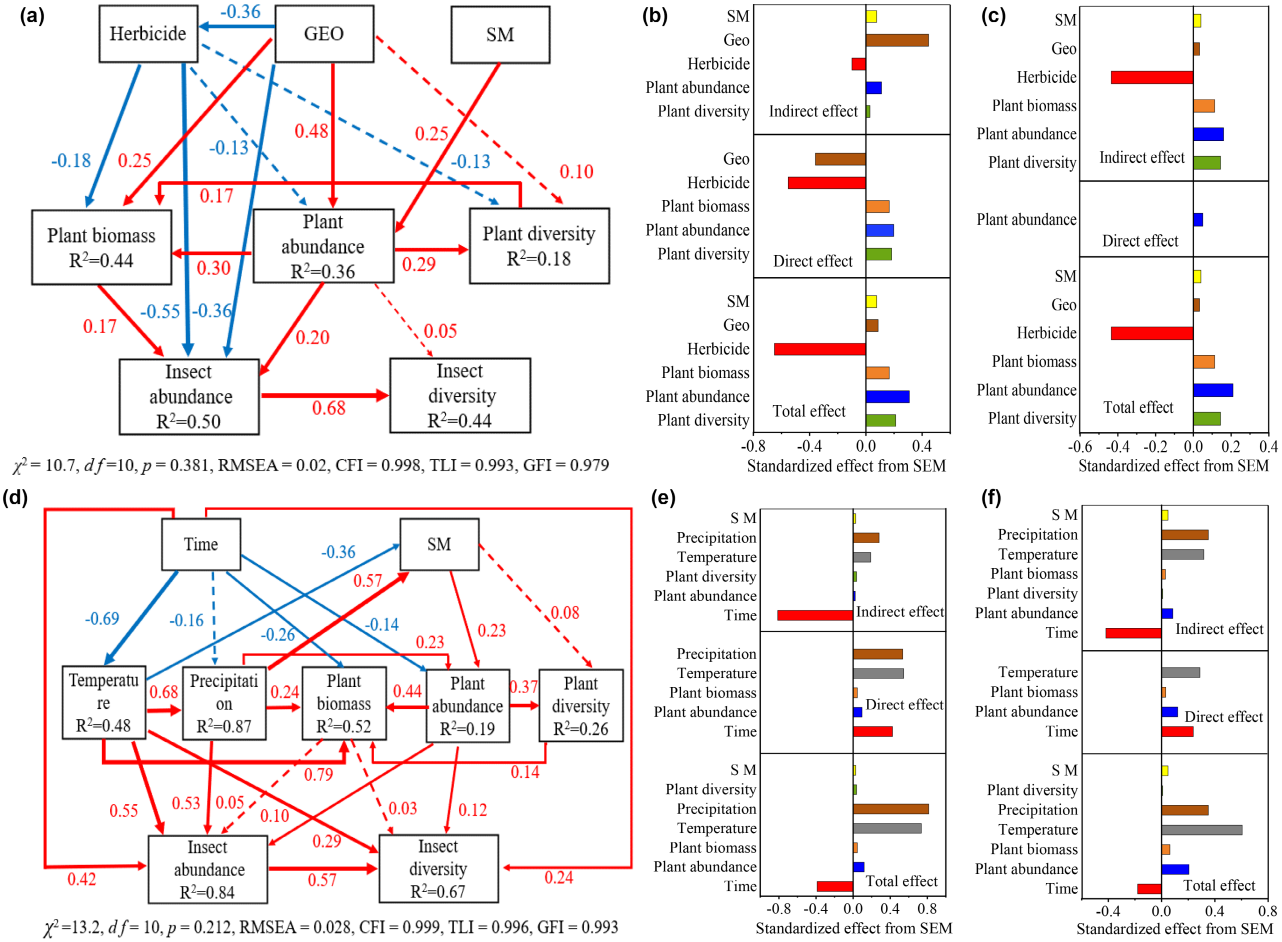
Structural equation modeling (SEM) of gully environmental factors influencing the insect diversity during herbicide application (a–c) and during the entire sampling stages (d–f). Solid red and blue arrows indicate significantly positive and negative relationships, respectively (*p* < .05); dashed arrows indicate nonsignificant relationships (*p* > .05); the thicker line represent the influence of factors on insects was stronger. SM, soil moisture; GEO, geographical location (represented by the position inside and outside the gullies); Time, crop growth stages; *χ* ^2^, chi‐square; *df*, degrees of freedom; *p*, probability level; CFI, comparative fit index; TLI, Tucker Lewis index; GFI, goodness of fit index; RMSEA, root mean square error of approximation.

Herbicide applications can directly reduce the abundance of insects in different SNH (Sharma et al., 2018; Zhang, Zhang, et al., 2023a). In this study, the aggregation of insects intensified after herbicide application, especially in stable gullies (Figure 2k,n). However, the richness and abundance ratios of BI/Pest were lower in gullies than in farmland after herbicide application before July. This means that many of the pest population may migrated from the farmland to the gullies' environment during the herbicide application. This also suggests that gully habitats provide a better refuge and food source for insects after herbicide application, particularly in terms of pest abundance. Additionally, our study identified herbicides indirect effects on insect diversity (Figure 8a), demonstrating that herbicides indirectly affect insect diversity by altering plant diversity and biomass. Notably, increased plant diversity in gullies positively correlates with insects' diversity in farmland (Zhang et al., 2016; Zhang, Zhang, et al., 2023a). However, both GLM and SEM analyses showed significant negative herbicide effects on functional insects, and the influence of herbicides was greater from the direct path than the indirect path on insect abundance, while it was the opposite on insect diversity (Figure 7a,b; Figure 8b,c). In addition, compared with the previous studies, the directly negative influences of herbicides on insect diversity were relatively higher (Norris & Kogan, 2000; Sonoda et al., 2011). This suggests that although herbicides are employed to manage weeds in farmland, both the indirect and direct impacts on functional insect populations cannot be overlooked, with the direct effects showing a tendency to intensify.

#### 4.1.2 Insect diversity influenced by climatic factors

In this study, insect diversity was primarily positively affected by precipitation and temperature in both gullies and farmlands during crop growth stages, and SEM analysis showed that insect diversity decreased with increasing growth stage (Figure 7c,d; Figure 8d–f). Insect diversity influenced mainly directly by temperature and indirectly by precipitation (Figure 8d). This indirect influence of precipitation is attributed mainly to its enhancement of the diversity of phytophagous beetles through the augmentation of plant abundance and the leaf traits functional diversity (Njovu et al., 2021). In addition, the number of reproductive generations of insects is impacted by temperature and food resources (natural plants), thus insect and plant resources often exhibit coevolution (Luo et al., 2011; Wen, 2010). It is noted that the dynamics of insects with plant growth were similar between gullies and the areas surrounding the gullies, which indicates that the diversity of insects is mainly influenced by seasons (the dynamics of temperature and precipitation) and plant growth.

#### 4.1.3 Insect diversity influenced by gullies

Compared to farmland, the richness and abundance of insects were 2.6 and 2.4 times higher in gullies after crop harvesting and the abundance ratios of BI/Pest was higher in this stage than without herbicide application phases (Figure 4a,b). This suggests that crop harvesting activities significantly impacted insect dynamics in farmland, likely due to habitat destruction, reduction of food sources, and direct harm to insects (Reberg‐Horton et al., 2011). Moreover, insects' richness and abundance in farmland were observed to be higher during periods without herbicide application and crop harvesting compared to after these events (Figure 2). This indicates a possible tendency for insects to migrate from gullies to nearby farmlands in search of abundant food sources and lower disturbance levels during the crop growth season (Schmidt et al., 2008). Interestingly, compared with other stages, the abundance ratios of BI/Pest were lower in both gullies and farmland during the vigorous crop growth stage, and the abundance of pests increased, especially in the farmland (Figure 4a,b). It is noted that the relatively higher abundance of crop pests tended to seriously affect crop growth and further pose a threat to crop yields. However, at this stage, the richness and abundance ratios of BI/Pest were greater in gullies than in farmlands, thus the gully as a source of BI was helpful in regulating the pests in farmland, further reducing the threat of pests to crops in farmland.

### 4.2 Dynamics of functional insects in single gullies

In this study, we found that the dynamic of abundance ratio of BI/pest remained almost consistent in all gully positions and farmland during all investigating periods (Figure 4e,f). The richness and abundance ratios of BI/Pest were mostly lowest in farmlands than in all gully positions (Figure 4e–h), while the abundance ratio of BI/Pest in the gully middle was highest than in other gully positions. This means that the dynamics of the ratio of BI/Pest in farmland could be mainly determined by the gullies, and the landscape element of gullies was beneficial to alleviate the negative effects of the pest on farmland crops especially for the middle positions of gullies. In addition, insects' richness and abundance were typically greater in gully middle than gully head and gully tail (Figure 5). This can be attributed to the larger area and soil moisture in gully middle, further leading to higher plant diversity (Table 1; Zhang, Zhang, et al., 2023a). Consistent with the findings from SEM analysis, soil moisture exhibited a positive correlation with insect diversity, with its influence on insect diversity being primarily mediated indirectly through plant abundance (Figure 8f). Furthermore, beyond climatic factors, plant abundance and biomass were identified as the most significant determinants of insect diversity (Figures 7c,d and 8e,f). Thus, the higher plant diversity and biomass in the gully middle with the higher soil moisture can feed more insects and provide a comfortable living condition, which also indicated that the gully middle was a key position for increasing both BI and pest, and the high abundance of insects with a relatively stable and high ratio of BI/Pest also performed the role of pests' biocontrol for the farmland.

Interestingly, following herbicide application, gully type has a significant effect on the abundance and richness of functional insects (Figure 7a,b). Alao, insect abundance and richness reached their peak in gully head in stable gullies, while in developing gullies, the highest values were observed in the gully middle. This may be due to the relatively deeper and better vegetation recovery in gully head compared to other gully positions, which was the best refuge for insects in the stable gullies (Morgan, 2005; Zhang, Zhang, et al., 2023a). In developing gullies, the highest insect populations were observed in the gully middle, likely due to constant erosion, scant vegetation, and collapse in gully head compared to other gully positions. Thus, the gully head served as the primary refuge for insects following herbicide application aimed at weed control in farmland in stable gullies. However, in developing gullies, the gully middle remained the predominant refuge for insects (Zhang, Zhang, et al., 2023a). Furthermore, the difference of insect's richness and abundance between any gully positions and farmland was greatest after herbicide application than in other crop phases (Figure 5). This indicates that herbicide application significantly affects insect distribution at both the watershed and single gullies scale (Sonoda et al., 2011; Zhang, Zhang, et al., 2023a).

### 4.3 Functional insects interspecific association in the watershed and single gullies

Interspecific association is a key indicator of a community's successional state and stability, as well as the relationship between species spatial distribution and environmental factors (Haak et al., 2020; Yang et al., 2021). Negative associations among species suggest that the community is in an earlier or secondary successional phase, along with lower interspecific association levels (Jin et al., 2022; Zhang, Zhang, et al., 2023a). In this study, we found that interspecific associations within functional insect communities were significant positive in the watershed, both during the crop bloom period and after herbicide application (Table 2). Further analysis, utilizing the chi‐square test, AC index and SRCC values, revealed that number of species‐pairs was usually greater in the presence of positive associations compared to negative ones. However, the functional insects interspecific association communities were not significant positive in single gullies, and number of species‐pairs was greater in negative associations than in positive ones (Figure S2; Tables 5, 6). These findings suggest that functional insect communities exhibit a higher degree of stability and interspecific association in the watershed, in contrast to the patterns observed in single gullies.

In addition, with negative correlations between species‐pairs in small scales and positive correlations between species‐pairs in larger watershed scales (Hortal et al., 2010; Liu et al., 2018; Zhang, Zhang, et al., 2023a). Our findings also revealed that number of species‐pairs was greater for negative correlation species in single gullies scale compared to the watershed scale. This could be attributed to interspecific relationships in single gullies being predominantly influenced by environmental factors and species interactions (Gerling et al., 2019; Zhang, Zhang, et al., 2023a). Thus, interspecies interactions and habitat heterogeneity were not only important in BI interspecies relationship formation, but also in the interspecies relationship formation in functional insects. Moreover, combining niche overlap with interspecific associations provides a more accurate representation of these relationships, with higher niche overlap indicating greater similarity in resource utilization by species (Lu et al., 2020). Our analysis also showed that niche overlap values between functional insects were relatively greater at both single gully and watershed (Tables 3, 4, 5, 6). The number of species‐pairs with a niche overlap value greater than 0.5 was more pronounced in single gullies than watershed (Tables 3, 5), suggesting that these species have consistent habitat requirements and that environmental resources are efficiently utilized in single gullies. Additionally, compared to watershed scales, niche overlap among species was higher in the single gully, where a stronger negative correlation in species–pairs was observed. This implies that habitat heterogeneity may enhance both intra‐ and interspecies competition among functional insect species in single gullies.

## 5 CONCLUSIONS

Stable gullies with higher plant diversity provide the best habitat for functional insects rather than forest belts and grassland in the watershed, especially during busy farming activities. Functional insects richness and abundance in farmland were determined by the gullies, especially in gully middle, and insect composition in farmlands influenced by stable gullies was stronger than developing gullies. Also, the vigorous growth stage of crop was one key period for pest control, and gully as a source of BI can significantly regulate the pests in farmland, further reducing the threat of pests to crops in this stage, especially in the position of middle stable gullies. In both the watershed and single gullies scales, functional insect dynamics were determined by season, followed by plant abundance and biomass in the gullies, and rarely by soil moisture.

## AUTHOR CONTRIBUTIONS

**Haijun Zhang:** Conceptualization (equal); data curation (lead); formal analysis (lead); investigation (lead); methodology (lead); software (lead); writing – original draft (lead); writing – review and editing (lead). **Shaoliang Zhang:** Conceptualization (supporting); formal analysis (equal); methodology (equal); resources (supporting); supervision (lead); writing – review and editing (equal). **Chengbo Zhang:** Investigation (supporting); software (supporting). **Ziliang Xiao:** Investigation (equal); supervision (equal). **Pengke Yan:** Investigation (equal); supervision (equal). **Muhammad Aurangzeib:** Supervision (supporting); writing – review and editing (supporting).

## CONFLICT OF INTEREST STATEMENT

The authors declare that there are no conflicts of interest.

## Supporting information


Data S1

Data S2

## Data Availability

The data that support the findings of this study are available in the supporting information of this article.
