# Supplementary material for: Stable gullies provide a suitable habitat for functional insects and reduce the threat of pests on crops in farmland of Northeast China
Source: Ecol Evol. 2024 Jul 7;14(7):e11686. doi: 10.1002/ece3.11686 (PMC11227938; doi:10.1002/ece3.11686)
Supplement: Supplementary file 1 — Data S1 [file ECE3-14-e11686-s002.docx]

**The alpha-diversity and interspecific association indices were calculated with the formula below:**

Shannon - Wiener (*H*)

$$\begin{aligned} H=-\sum\left( P_{i} \right)\left( \ln P_{i} \right) \left( 1 \right) \end{aligned}$$

Where *Pi=S_i_/S* means the number proportion of species *i* to all species.

Species importance value (*IV*)

$$\begin{aligned} IV=\left( RA+RF \right)/2 \left( 2 \right) \end{aligned}$$

Where *RA* is the relative abundance, the proportion of individuals of the species. *RF* is the relative frequency, the ratio of the frequency of the species in all samples to the sum of the frequencies of all species. The sum of the important values of all species is 1.

Niche overlap (*O_ik_*) (Pianka, 1973)

$$\begin{aligned} O_{ik}\text{=}{\sum_{j=1}^{r} P_{ij}P_{kj}}/{\sqrt{\left( \sum_{j=1}^{r} P_{ij} \right)^{2}\left( \sum_{j=1}^{r} P_{kj} \right)^{2}}}\text{ }\text{(3)} \end{aligned}$$

Where *P_ij_* and *P_kj_* are the proportion of the number of species *i* and species *k* to the number of all species in sample *j*, and *r* is the number of samples.

Variance ratio (*VR*)（Schluter, 1984)

$$\begin{aligned} VR={S_{T}^{2}}/{\delta_{T}^{2}}={\frac{1}{N}\sum_{i=1}^{N} \left( T_{j}-t \right)^{2}}/{\sum_{i=1}^{S} P_{i}\left( 1-P_{i} \right)} \left( 4 \right) \end{aligned}$$

$$\begin{aligned} W=VR\times N \left( 5 \right) \end{aligned}$$

Where $S_{T}^{2}$ is the variance of the number of species in all samples, $\delta_{T}^{2}$ is the variance of the frequency of occurrence of all species, *S* is the total number of species, *N* is the total number of samples, *P_i_* is the frequency of occurrence of species *i*, *P_i_ =n_i_/N*, *n_i_* is the number of samples in containing species *i*, *T_j_* is the number of species occurring in sample *j*, *t* is the mean number of species in the samples. When *VR*>1, the species were positively associated. When *VR*<1, the species were negatively associated. When *VR*=1, the species were non-associated. Also, we used the *W* statistic value to further test for significance. If overall interspecific associations were not significant (*P* > 0.05), the *W* values were within *χ^2^* critical value (*χ^2^_0.95(N)_< W <χ^2^_0.05(N)_*) with 90% probability, otherwise, the association was significant (*P* < 0.05).

Chi-square test (*χ^2^*)

$$\begin{aligned} \chi^{2}={N\left[ \left| ad-bc \right|-\left( \frac{N}{2} \right) \right]^{2}}/{\left( a+b \right)\left( a+c \right)\left( b+d \right)\left( c+d \right)} \left( 6 \right) \end{aligned}$$

Where *N* is the total number of samples, *a* is the abundance of samples where both species are found, *b* and *c* are the number of samples where both species are found alone, and *d* is the number of samples where neither species is found.

Association coefficients (*AC*)

$$\begin{aligned} ad\geq bc AC=\left( ad-bc \right)/\left[ \left( a+b \right)\left( b+d \right) \right] \left( 7 \right) \end{aligned}$$

$$\begin{aligned} ad<bc,a\leq d AC=\left( ad-bc \right)/\left[ \left( a+b \right)\left( a+c \right) \right]\left( 8 \right) \end{aligned}$$

$$\begin{aligned} ad<bc,a>d AC=\left( ad-bc \right)/\left[ \left( b+d \right)\left( d+c \right) \right] \left( 9 \right) \end{aligned}$$

Where *a*, *b*, *c*, and *d* are the same as above. The closer the *AC* value is to 1, the stronger the positive correlation between the species. The closer the *AC* value is to −1, the stronger the negative correlation between the species. If the *AC* value is 0, the two species are entirely independent.

Spearman rank correlation coefficient (*r_p_*)

$$\begin{aligned} r_{p}\left( i,k \right)=1-6{\sum_{j=1}^{N} \left( x_{ij}-\bar{x}_{i} \right)^{2}\left( x_{kj}-\bar{x}_{k} \right)^{2}}/{(N^{3}-N)} \left( 10 \right) \end{aligned}$$

Where *r_p_ (i, j)* is the Spearman rank correlation coefficient between species *i* and species *k* in sample *j*, respectively. *N* is the total abundance of samples, *x_ij_* and *x_kj_* are the rank of the number of species *i* and species *k* in sample *j*, respectively.

**Table S1.** Description of the main plant species investigated in the main habitats of the watershed.

| Habitats | Plant species | Pollination medium | Growth cycle | Blooming stage |
| --- | --- | --- | --- | --- |
| Gully | Elymus dahuricus | entomophily | perennial | July-September |
|  | Artemisia lavandulifolia | anemophily | perennial | August-October |
|  | Equisetum arvense |  | perennial |  |
|  | Artemisia caruifolia | anemophily | annual | June-September |
|  | Calamagrostis epigeios | anemophily | perennial | July-August |
|  | Geranium wilfordii | entomophily | perennial | June-August |
|  | Festuca glauca | anemophily | perennial | May-October |
|  | Aster hispidus | entomophily | annual or biennial | July-September |
|  | Poa pratensis | entomophily | perennial | May-August |
|  | Cirsium setosum | entomophily | perennial | June-September |
|  | Phragmites australis | anemophily | perennial | August-October |
|  | Vicia hirsuta | anemophily | annual | July-September |
|  | Scutellaria baicalensis | entomophily | perennial | July-August |
|  | Galium verum | entomophily | perennial | July-August |
| Peak of blooming | Chenopodium album | Anemophily | Annual | May-October  **July-August** |
| Farmland | Equisetum arvense |  | perennial |  |
|  | Echinochloa crusgalli | anemophily | annual | July-August |
|  | Zea mays | anemophily | annual | July-August |
|  | Cirsium setosum | entomophily | perennial | June-September |
|  | Chenopodium album | anemophily | annual | May-October |
| Peak of blooming |  |  |  | **July-August** |
| Grassland | Elymus dahuricus | entomophily | perennial | July-September |
|  | Chenopodium album | anemophily | annual | May-October |
|  | Equisetum arvense |  | perennial |  |
|  | Cirsium setosum | entomophily | perennial | June-September |
|  | Echinochloa crusgalli | anemophily | annual | July-August |
| Peak of blooming |  |  |  | **July- September** |
| Forest belt | Artemisia caruifolia | anemophily | annual | June-September |
|  | Elymus dahuricus | entomophily | perennial | July-September |
|  | Equisetum arvense |  | perennial |  |
|  | Aster hispidus | entomophily | annual or biennial | July-September |
|  | Phragmites australis | anemophily | perennial | August-October |
| Peak of blooming |  |  |  | **July- September** |


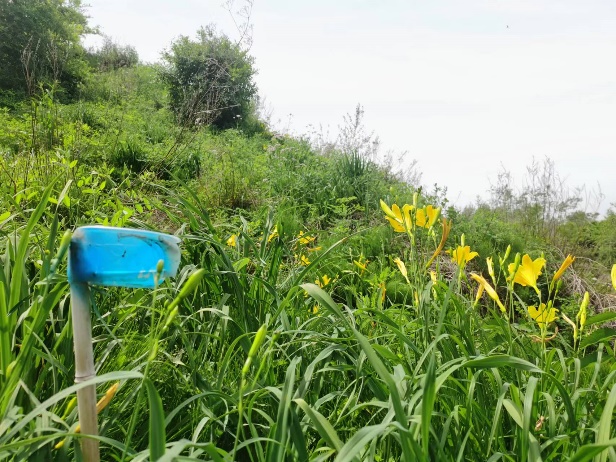

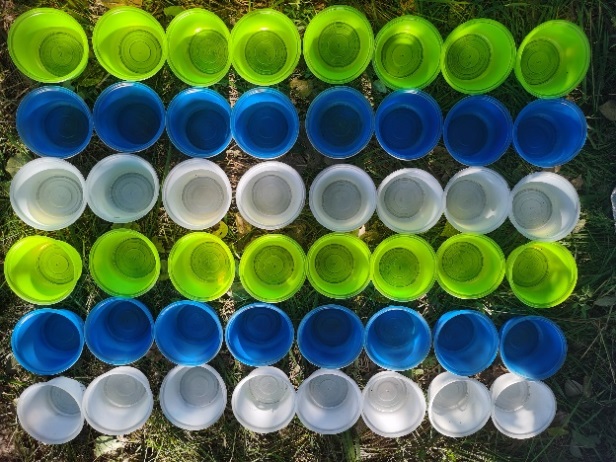

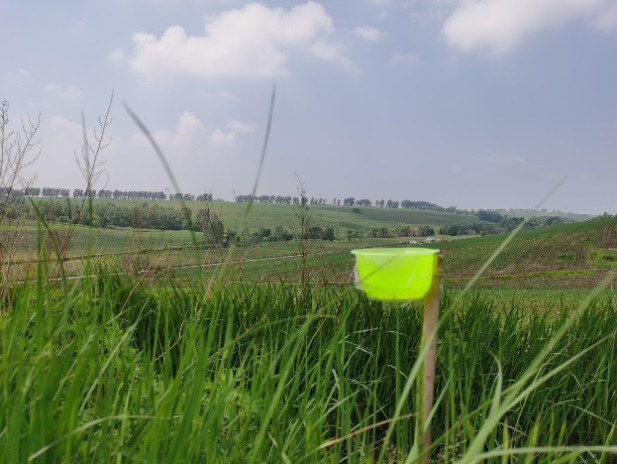

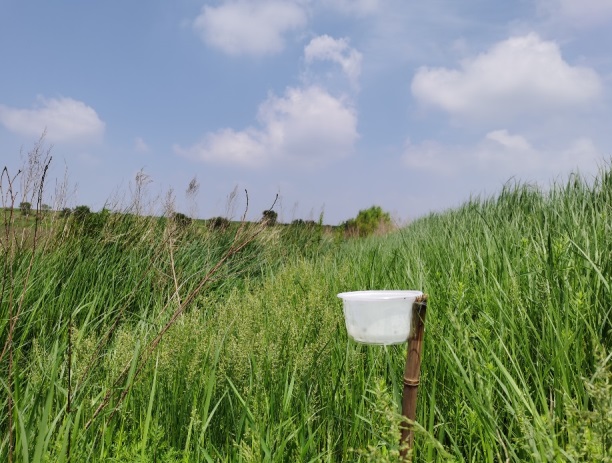

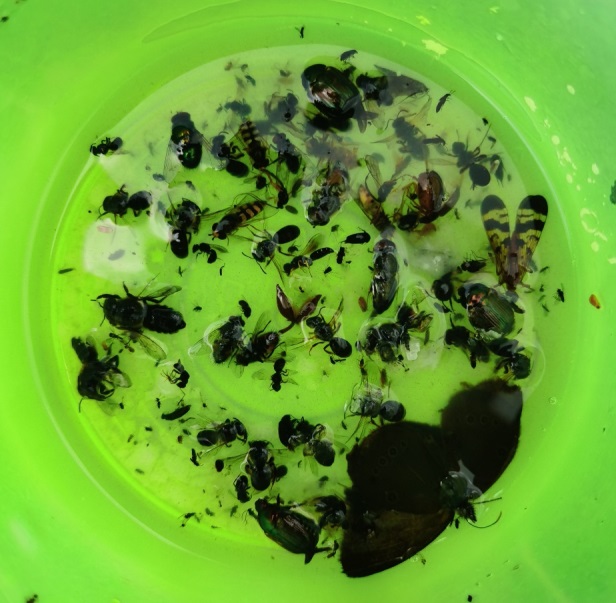

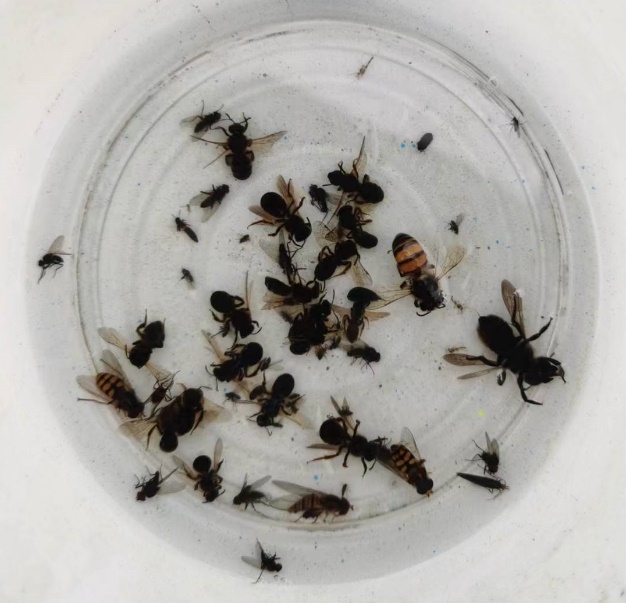


**Fig. S1.** The equipment of insect collection in the field


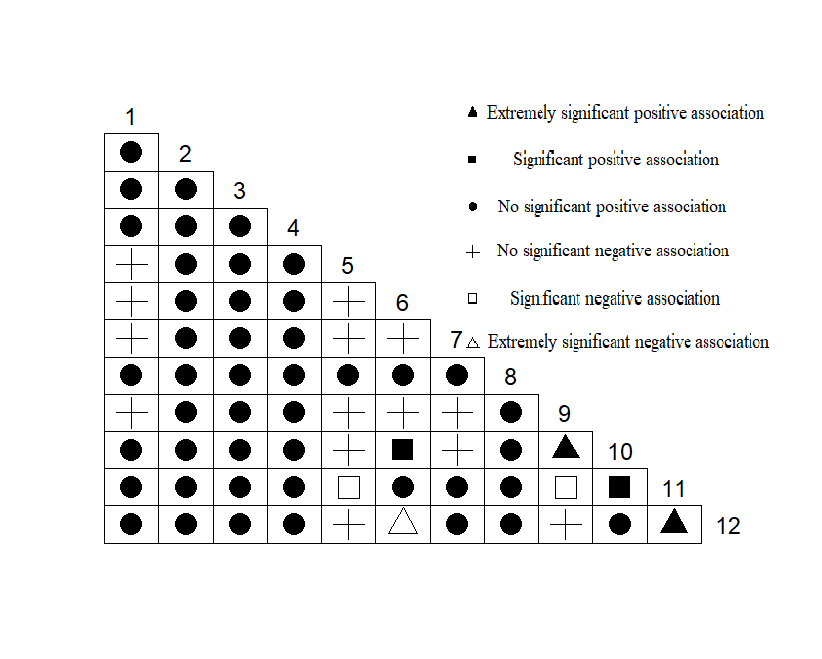

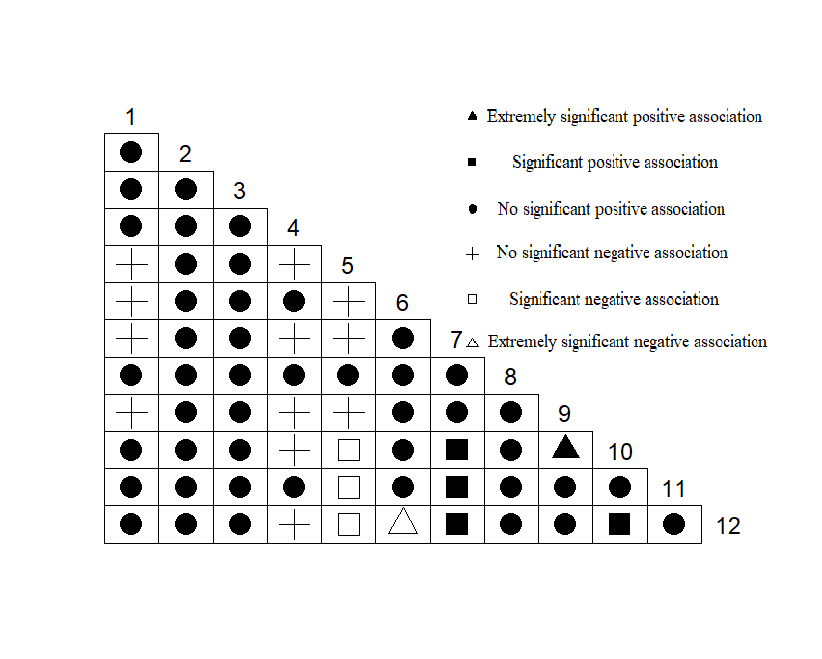

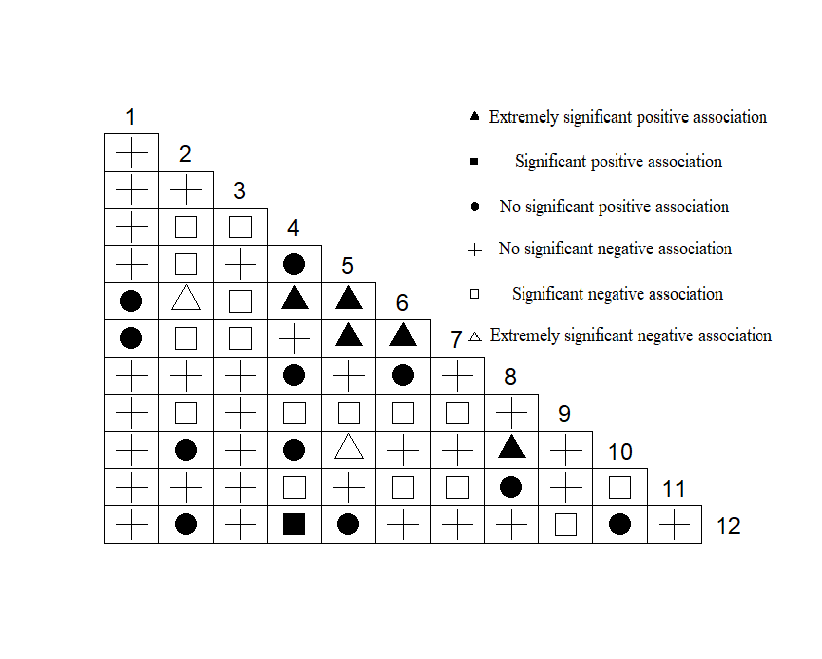

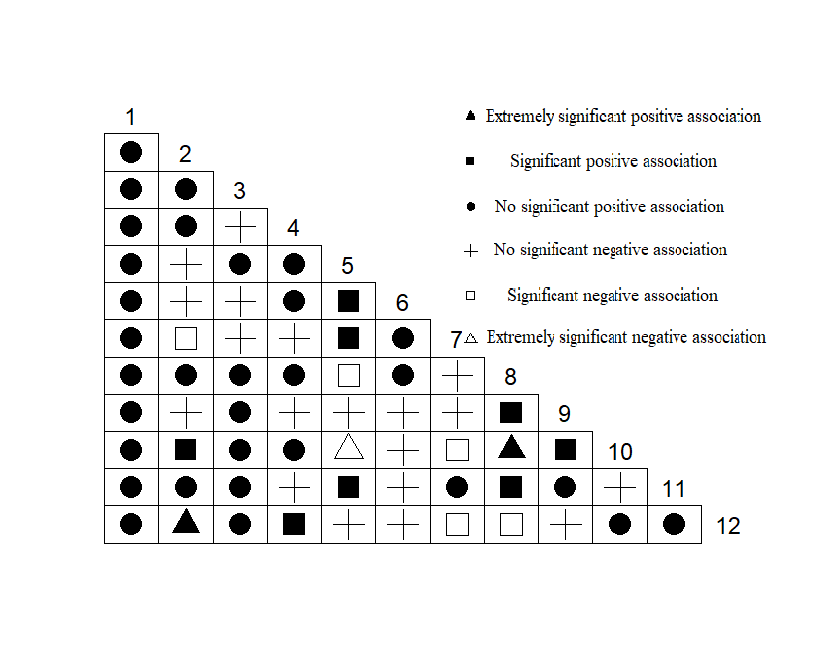


a

b

c

d

**Fig. S2.** Chi-Square test between species-pairs after herbicide application (a) and during flowering (b) in watershed. Chi-Square test values between species-pairs after herbicide application (c) and during flowering (d) in single gullies. Sub-figure a and c the serial number from 1 to 12 represents the insect species of Formicidae, Delphacidae, Chrysomelidae, Pyralidae, Bibionidae, Cicadidae, Apidae, Muscidae, Acridoidea, Carabidae, Syrphidae, Ceratopogonidae. Sub-figures b and d the serial number from 1 to 12 represents of Chrysomelidae, Gryllidae, Formicidae, Muscidae, Delphacidae, Acridoidea, Pyralidae, Meloidae, Apidae, Scarabaeidae, Panorpidae, Carabidae.


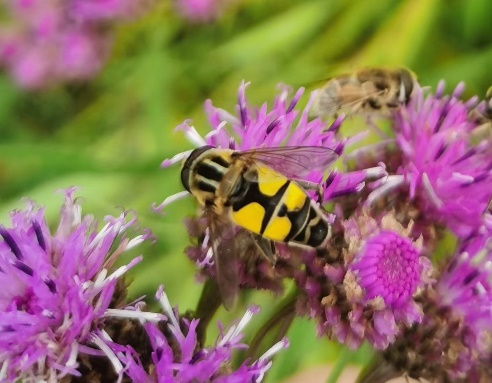

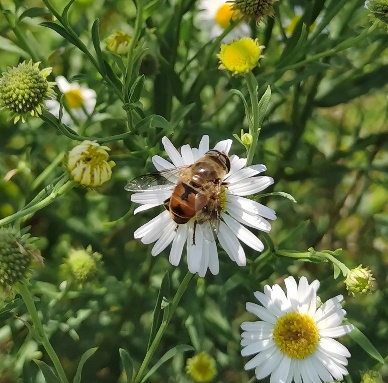

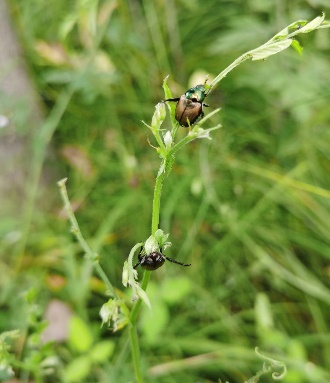


a

b

c

**Fig. S3**. Functional insect-plant interactions. Sub-figure a represents Helophilus fasciatus and Saussurea japonica. Sub-figure b represents Eristalis cerealis and Aster hispidus. Sub-figure c represents Anomala corpulenta Motschulsky and Vicia hirsute.


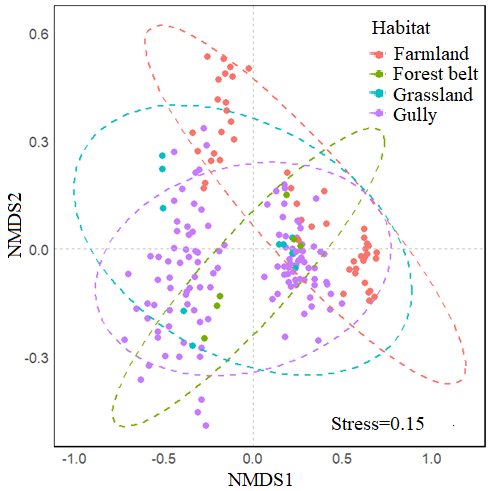


**Fig. S4**. β-diversity of functional insects within different habitats in the watershed.

**Fig. S5**. Proportion of different functional insect abundance (a) and richness (b) in the study area.


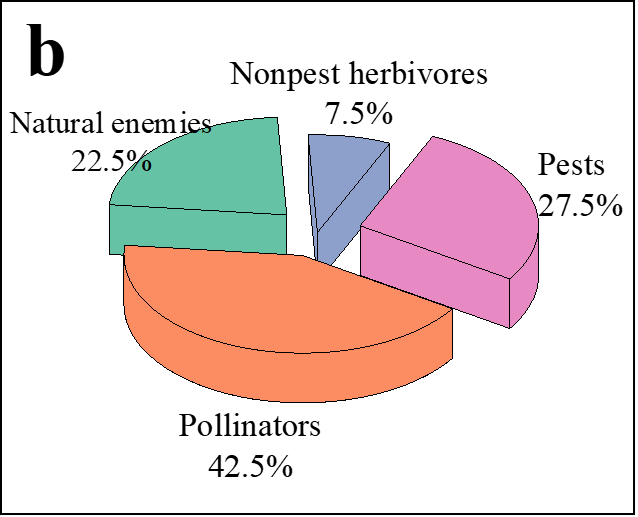

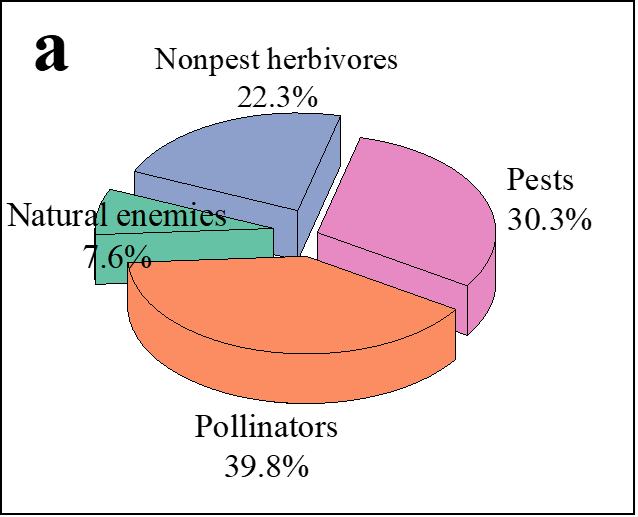


**References**

Pianka, E.R., 1973. The structure of lizard communities. Annu. Rev. Ecol. Syst. 4, 53-74

Schluter, D., 1984. A variance test for detecting species associations, with some example applications. Ecology 65, 998-1005.

Zhang, S.L., Wang, X.R., Xiao, Z.L., Qu, F.J., Wang, X.S., Li, Y., Aurangzeib, M., Zhang, X.Y., Liu, X.B., 2020. Quantitative studies of gully slope erosion and soil physiochemical properties during freeze-thaw cycling in a Mollisol region. Sci. Total Environ. 707, 136191.
